# The Role of Mg(OH)_2_ in the So‐Called “Base‐Free” Oxidation of Glycerol with AuPd Catalysts

**DOI:** 10.1002/chem.201704151

**Published:** 2018-01-24

**Authors:** Jile Fu, Qian He, Peter J. Miedziak, Gemma L. Brett, Xiaoyang Huang, Samuel Pattisson, Mark Douthwaite, Graham J. Hutchings

**Affiliations:** ^1^ Cardiff Catalysis Institute School of Chemistry Cardiff University Main Building, Park Place Cardiff CF10 3AT United Kingdom; ^2^ Department of Chemical and Biochemical Engineering College of Chemistry and Chemical Engineering National Engineering Laboratory for Green Chemical Productions of Alcohols-Ethers-Esters Xiamen University Xiamen 361005 P. R. China

**Keywords:** base-free, gold, magnesium, nanoparticles, oxidation

## Abstract

Mg(OH)_2_‐ and Mg(OH)_2_‐containing materials can provide excellent performance as supports for AuPd nanoparticles for the oxidation of glycerol in the absence of base, which is considered to be a result of additional basic sites on the surface of the support. However, its influence on the reaction solution is not generally discussed. In this paper, we examine the relationship between the basic Mg(OH)_2_ support and AuPd nanoparticles in detail using four types of catalyst. For these reactions, the physical interaction between Mg(OH)_2_ and AuPd was adjusted. It was found that the activity of the AuPd nanoparticles increased with the amount of Mg(OH)_2_ added under base‐free conditions, regardless of its interaction with the noble metals. In order to investigate how Mg(OH)_2_ affected the glycerol oxidation, detailed information about the performance of AuPd/Mg(OH)_2_, physically mixed (AuPd/C+Mg(OH)_2_) and (AuPd/C+NaHCO_3_) was obtained and compared. Furthermore, NaOH and Mg(OH)_2_ were added during the reaction using AuPd/C. All these results indicate that the distinctive and outstanding performance of Mg(OH)_2_ supported catalysts in base‐free condition is in fact directly related to its ability to affect the pH during the reaction and as such, assists with the initial activation of the primary alcohol, which is considered to be the rate determining step in the reaction.

## Introduction

Biomass conversion has drawn worldwide attention in recent years because of its potential to be an alternative resource to fossil fuel. The gradually increasing production of biodiesel has generated a huge amount of by‐products, the major part of which is glycerol.^1^ It is reported that glycerol is comprised of approximately 10 wt % of the total product yield for biodiesel production^2^ and as such, it is projected that the demand for the consumption of glycerol will increase from 2000 kt in 2011 to 3070 kt by 2018.^3^ Therefore, transforming glycerol into valuable derivatives could be an effective method to increase the economic efficiency of biodiesel production.

In the past decades, great improvements have been made on the catalytic oxidation of glycerol. Gold‐supported catalysts were found to be efficient for the conversion of glycerol to C_3_ products under basic conditions.^4^ When compared with Pd, Pt and other noble metals, gold is more resistant to the formation of oxides^5^ which can poison the catalyst, making gold highly active, selective and stable. In subsequent studies, alloyed Au−Pd,^6^ Au−Pt^7^ and Au−Pt−Pd^8^ nanoparticles, prepared by a colloidal approach, demonstrated much better performance than corresponding monometallic catalysts. However, the requirement of base addition for gold‐based catalysts in glycerol oxidation is not favourable for industrial applications, since the products are salts of acids, which then need further treatment. Thus, considerable attention has been focused on the investigation of glycerol oxidation under base‐free conditions. Prati and co‐workers investigated the performance of H‐mordenite‐supported alloyed Au−Pt nanoparticles compared with the same particles supported on activated carbon, MgO, SiO_2_, ZrO_2_ and MCM‐41.^9^ Among these, MgO‐supported catalysts exhibited superior activity, however, the selectivity to C_2_ or C_1_ products was very high. By using a modified colloidal methodology and controlling the preparation temperature, the performance of the Au−Pd and Au−Pt on MgO or Mg(OH)_2_ could be significantly improved.^10^ The beneficial effect of supporting such particles on MgO and Mg(OH)_2_ was subsequently studied in depth and compared with other basic and acidic supports. It was reported that the quantity and strength of basic sites on MgO and Mg(OH)_2_ have a large influence on the product distribution.^11^ This influence was also observed with other basic supports such as (MgCO_3_)_4_Mg(OH)_2_ and CaCO_3_. Xu and co‐workers investigated the Au/Al_2_O_3_−MgO for glycerol oxidation.^12^ They found that the percentage composition of MgO could affect the acid‐base properties of the catalysts, resulting in different activities and product distributions. Similar results were also observed in the base‐free oxidation of 5‐hydroxymethylfurfural. Wang and co‐workers synthesized a mixed C‐O‐Mg support.^13^ It was suggested that the strong and stable basic sites of the support were the reason behind why such high activities were observed with these catalysts.

MgO is sparingly soluble in water (86 mg L^−1^ at 30 °C)^14^ and the products of glycerol oxidation are often organic acids. Some previous publications have revealed that metals from solid basic supports can leach into aqueous solution when no additional base is added to the reaction,^10^, ^15^ a phenomena which has shown to be suppressed at elevated pH′s.^16^ Given that it is known that these materials can partially dissolve in an aqueous media, it is somewhat surprising that no attempts have been made to correlate this with the perceived increase in catalytic performance observed with solid basic supports. This is especially important given that pH has been shown to have such a significant effect on the rate‐determining step in the oxidation of alcohols.^17^

It is clear that MgO or Mg(OH)_2_ plays a key role in the performance of Au, Au−Pd and Au−Pt nanoparticles under base‐free conditions. Most of the previous investigations were focussed on the properties of basic sites on the surface of the supports, which were correlated with the activity and selectivity of catalysts. However, no detailed information, regarding the effect of the solid base on the reaction conditions or its effect on the performance of catalysts was provided. In this work, the catalytic behaviour of Au−Pd nanoparticles on different types of support was investigated. The catalytic performance is found to be directly related to the pH of the reaction solution, which could be altered by the support. The aim of this paper is to provide a new perspective on how basic supports, such as Mg(OH)_2_, affect the activity and selectivity of Au based catalysts in the oxidation of glycerol by careful consideration and monitoring of the pH of the solutions during glycerol reactions. This pH change can be mimicked with the addition of a sacrificial base and the results correlated.

## Results and Discussion

### The performance and structure of AuPd−Mg(OH)_2_/C

AuPd−Mg(OH)_2_/C catalysts with varying quantities of Mg(OH)_2_ were prepared by the sol‐immobilization method using the pre‐synthesized support Mg(OH)_2_/C. These catalysts were observed to be effective for glycerol oxidation under “base‐free” conditions (Supporting Information Figure S1) when high amounts of Mg(OH)_2_ were loaded onto the carbon support. It has been previously reported that a mixed carbon/Mg(OH)_2_ support shows much better activity than carbon alone in 5‐hydroxymethylfurfural (HMF) oxidation with Pt nanoparticles under base‐free reaction conditions, which has been ascribed to the basic sites provided by Mg^2+^.^13^ Therefore, in view of these results we reasoned that the AuPd−Mg(OH)_2_/C should be present as a triple layer composite where the AuPd nanoparticles were selectively located on the Mg(OH)_2_, thereby enabling the interaction between basic sites and noble metals. Subsequently, to determine whether the synthesised catalyst has this triple layer structure, X‐ray diffraction (XRD) and transmission electron microscopy (TEM) characterization of the catalysts was performed.

**Figure 1:**
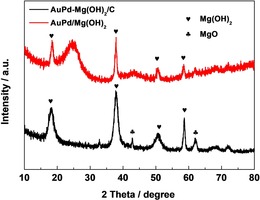
XRD patterns of AuPd/Mg(OH)_2_ and AuPd−Mg(OH)_2_/C.

**Figure 2:**
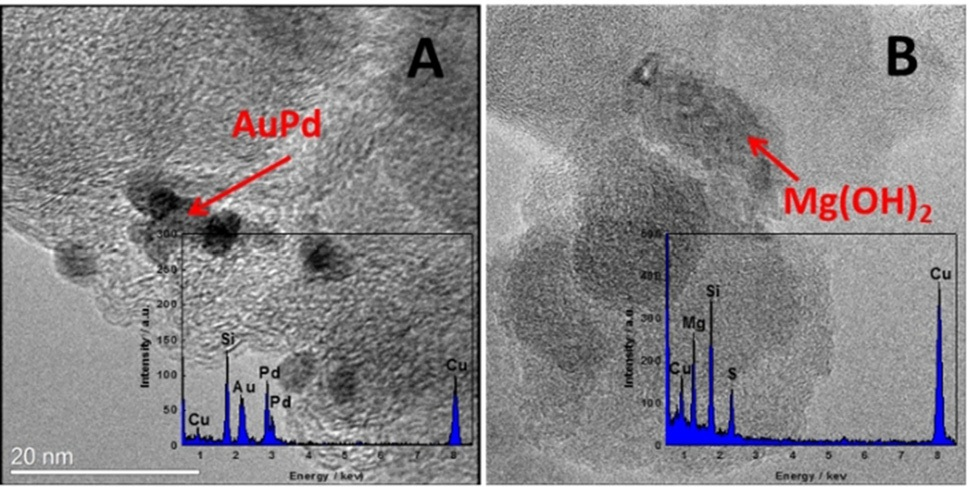
TEM and EDX of AuPd−Mg(OH)_2_/C. Graph A and Graph B show the surface information of different parts of AuPd−Mg(OH)_2_/C. It is apparent that AuPd nanoparticles and Mg(OH)_2_ are located on different areas of the catalyst.

The XRD analysis in Figure 1 for the AuPd−Mg(OH)_2_/C catalyst confirmed the presence of Mg(OH)_2_ on the carbon. No Au and Pd phase could be observed, which is ascribed to the low metal content and the effective dispersion of the AuPd nanoparticles. Figure 2 shows the TEM and energy‐dispersive X‐ray spectroscopy (EDX) results of AuPd−Mg(OH)_2_/C. It is clear that AuPd nanoparticles are located on the carbon without any interaction with the Mg(OH)_2_ (Figure 2 A). In Figure 2 B, Mg is observed as a separate phase. Thus, it could be concluded that AuPd and Mg(OH)_2_ were separately located on carbon for the catalyst AuPd−Mg(OH)_2_/C rather than forming the intended triple layer structure. As we did not form the triple layer structure, we investigated the sol‐immobilization process to compare the adsorption ability between XC‐72R and Mg(OH)_2_ separately and the time taken for the support to adsorb nanoparticles from the solvent. XC‐72R only needed 10 min to adsorb the AuPd nanoparticles, whereas Mg(OH)_2_ needed at least 30 min. Hence the carbon support has a much higher affinity for the adsorption of the nanoparticles and therefore, they will be preferentially located on the carbon support.

Previously it has been reported that the superior performance of Mg(OH)_2_‐containing catalysts is considered to be related to the basic sites that interact with the metal nanoparticles. However, AuPd−Mg(OH)_2_/C is able to achieve a very high activity without direct contact between AuPd and Mg(OH)_2_. Thus, we concluded that the role of the Mg(OH)_2_ in the oxidation of glycerol under “base‐free” conditions needed to be further considered.

### The performance of AuPd nanoparticles with different locations of Mg(OH)_2_

To further investigate the role of Mg(OH)_2_ in the oxidation reaction, we synthesised a series of additional catalysts. Au−Pd nanoparticles were deposited on MgO and carbon by the same sol‐immobilization method used for the preparation of the mixed‐support catalyst. From the XRD results shown in Figure 1, it is clear that MgO is converted to Mg(OH)_2_ during the catalyst preparation process through hydrolysis, which is consistent with our reported results.^10^, ^15^ As such, this catalyst is denoted as AuPd/Mg(OH)_2_.

**Figure 3:**
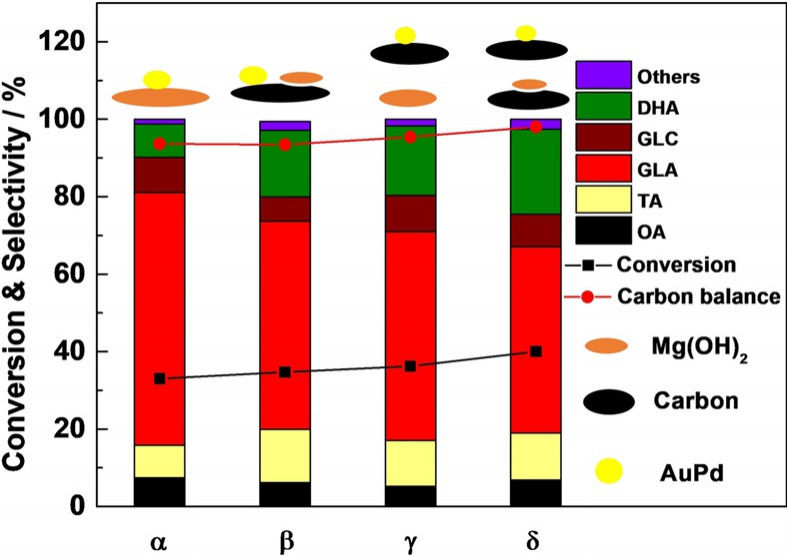
The effect of Mg(OH)_2_ location on the performance of the AuPd nanoparticles in “base‐free” glycerol oxidation. Four kinds of catalyst were tested, including *α* (AuPd/Mg(OH)_2_), *β* (AuPd−Mg(OH)_2_/C), *γ* (physically mixed AuPd/C+Mg(OH)_2_), and *δ* (physically mixed AuPd/C+Mg(OH)_2_/C). The Mg(OH)_2_/glycerol mole ratio is 0.34, except for AuPd−Mg(OH)_2_/C which is 0.23. OA (oxalic acid), TA (tartronic acid), GLA (glyceric acid), GLC (glycolic acid), DHA (dihydroxyacetone). Reaction conditions: 1:1.85 mole fraction Au:Pd with 1 % metal loading by mass, water (10 mL), glycerol (0.3 m), mole fraction glycerol/metal=690, 60 °C, 4 h, 3 bar O_2_.

The four types of catalyst (AuPd/Mg(OH)_2_, AuPd−Mg(OH)_2_/C, physically mixed (AuPd/C+Mg(OH)_2_) and physically mixed (AuPd/C+Mg(OH)_2_/C)) were subsequently tested for glycerol oxidation in the absence of sacrificial base. As we have shown in Figure 2, the AuPd and Mg(OH)_2_ are located separately on the carbon for AuPd−Mg(OH)_2_/C. Therefore, the interaction between metal nanoparticles and Mg(OH)_2_ decreases in these four catalysts, as the distance between the AuPd and Mg(OH)_2_ increases. However, as is shown in Figure 3, there is not a large difference among these four catalysts with respect to their performance for the oxidation of glycerol. AuPd/Mg(OH)_2_ even presents slightly lower activity (33 % conversion) when compared with the other carbon‐supported catalysts with Mg(OH)_2_ (from 36 to 40 %). With respect to the product distribution, AuPd/Mg(OH)_2_ gives a slightly lower selectivity towards dihydroxyacetone and tartronic acid, accompanied with a higher selectivity for glyceric acid. To demonstrate the superior activity of catalysts with Mg(OH)_2_; AuPd/C, AuPd/CeO_2_ and AuPd/TiO_2_ were also tested under the same conditions. They show an extremely low activity for glycerol oxidation and totally different product distributions are observed when Mg(OH)_2_ was not present (Figure S2). As such, we hypothesised that the effective performance of the AuPd nanoparticles could be obtained as long as Mg(OH)_2_ was added, regardless of its interaction with the nanoparticles and where the AuPd was located.

### Comparison between physically mixed (AuPd/C+Mg(OH)_2_) and AuPd/C with the addition of NaHCO_3_

**Figure 4:**
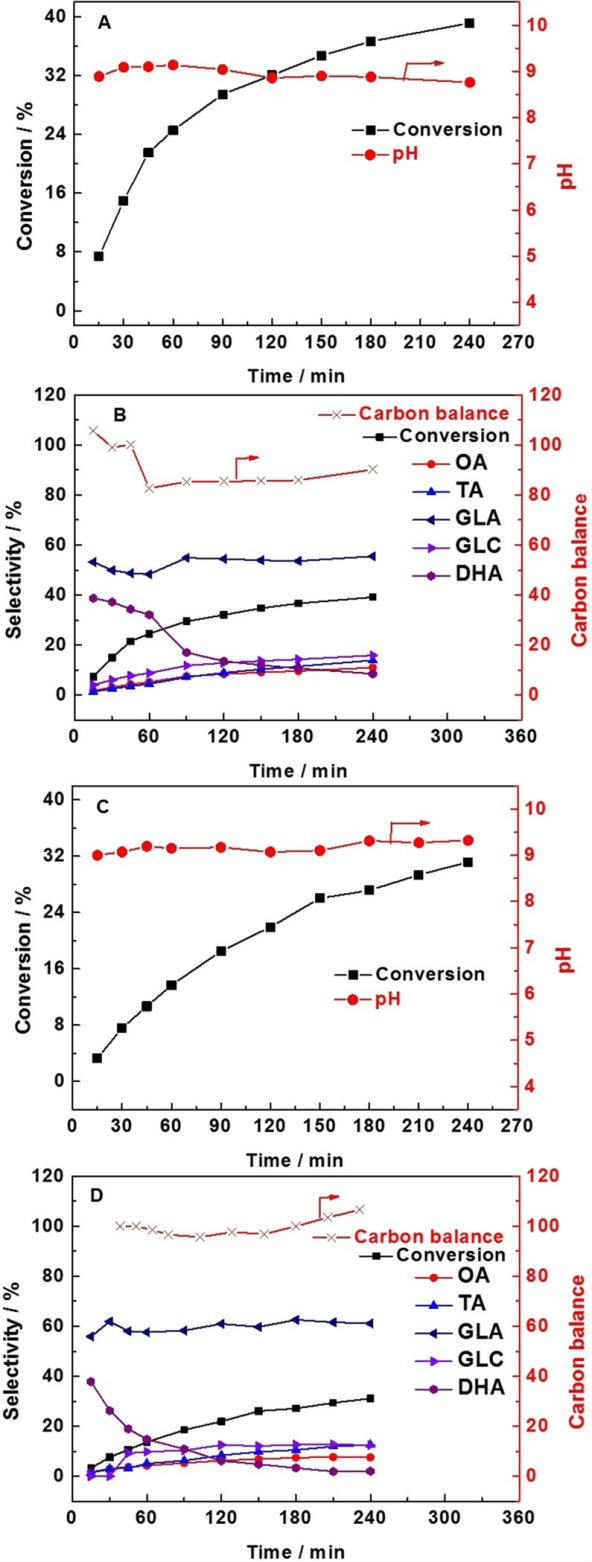
Conversion/selectivity/pH/carbon balance vs. time profiles of glycerol oxidation. OA (oxalic acid), TA (tartronic acid), GLA (glyceric acid), GLC (glycolic acid), DHA (dihydroxyacetone). Graph A and B present the results of physically mixed (AuPd/C+Mg(OH)_2_), Mg(OH)_2_/glycerol mole fraction=0.34. Graph C and D present the performance of (AuPd/C+NaHCO_3_), mole fraction NaHCO_3_/glycerol=3. Reaction conditions: 1:1.85 mole fraction Au:Pd with 1 % metal loading by mass, water (35 mL), glycerol (0.3 m), mole fraction glycerol/metal=690, 60 °C, 4 h, 3 bar O_2_.

Having established that the location of the MgO does not affect the conversion of glycerol, we decided to compare the effect of its presence to a sacrificial base. Initially, we obtained time online data for the physically mixed (AuPd/C+Mg(OH)_2_) catalyst with the pH of the solution recorded at each point (Figure 4 A,B). For the purpose of facilitating the comparison, the glycerol/Mg(OH)_2_ mole fraction was maintained at 0.34. It is clear, as expected, that the glycerol conversion increases gradually with time. The selectivity to dihydroxyacetone (DHA) declines continuously, while the selectivity to glycolic acid and oxalic acid increases slowly, the selectivity to tartronic acid shows a similar trend while the selectivity to glyceric acid reaches a steady state after 60 min. These results are in line with the reaction mechanism reported previously,^18^ as it is widely accepted that glycerol could be consecutively oxidized to form glyceraldehyde, glyceric acid and then tartronic acid. DHA and glyceraldehyde are the primary oxidation products of glycerol oxidation, and the transformation between them is considered to be reversible in alkaline media.^19^, ^20^ Since the oxidation of glyceraldehyde is very facile, no glyceraldehyde could be observed. DHA and tartronic acid can be further oxidized to glycolic acid and oxalic acid, respectively. Therefore, the gradual decrease of DHA could be ascribed to the formation of glycolic acid and glyceraldehyde which can subsequently undergo further oxidation to glyceric acid. The steady state observed for glyceric acid selectivity is likely to be a result of the rate of formation being comparable with the subsequent oxidation to tartronic acid. In addition to monitoring the glycerol conversion and product distribution, the pH of the solution was also monitored with time. Interestingly, the pH of the solution appeared to remain fairly constant at pH 9 for the duration of the experiments in both systems (Figure 4). The corresponding data sets for the reactions over the AuPd/Mg(OH)_2_ catalyst and AuPd/C+Mg(OH)_2_ system were found to be comparable with respect to the observed pH and product distribution. Slightly higher conversions were observed however, with the AuPd/C+Mg(OH)_2_ system.

The *K*_sp_ of Mg(OH)_2_ at 25 °C is 5.62×10^−12^, which means that the pH of water should be around 10.3 in the presence of Mg(OH)_2_. With the addition of glycerol and its acid products, OH^−^ would be continuously consumed. As a result, the pH would be expected to decrease and the concentration of Mg^2+^ in the solution would increase according to the solubility equilibrium and acid‐base dissociation equilibrium. The pH of the solution can be calculated from the Mg^2+^ concentration and *K*_sp_ of Mg(OH)_2_. The pH is estimated to be 9 for the solution with the AuPd/Mg(OH)_2_ catalyst after 4 h reaction on the basis of Mg concentration (1300 ppm). This value is consistent with the pH measured as shown in Figure S3 (Supporting Information). Therefore, the observed pH of the solution is consistent with the dissolution of Mg(OH)_2_ and the consumption of OH^−^ by the acids produced from glycerol oxidation.

To investigate the influence of pH on the performance of the AuPd nanoparticles, Mg(OH)_2_ was replaced by NaHCO_3_. As is shown in Figure 4 C, the conversion of glycerol increases gradually, while the pH is maintained at approximately pH 9, which is in accordance with the conversion observed in the presence of Mg(OH)_2_. After 4 h, the conversion reaches 31 %, which is similar to the results observed for the AuPd/Mg(OH)_2_ and physically mixed (AuPd/C+Mg(OH)_2_) systems. As for the evolution of products (Figure 4 D), glyceric acid was kept steady at arpproximately 60 % and the selectivity of DHA decreased continuously from 39 to <10 %. The selectivity to glycolic acid, oxalic acid and tartronic acid increased slowly during the process. It is apparent that a similar performance is observed for both catalysts when the pH is maintained at the same level (pH 9). Thus, it is logical to correlate the high performance of the Mg(OH)_2_‐supported catalysts with its ability to maintain the pH at a high level through the dissolution of Mg(OH)_2_.

### Effect of [Mg(OH)_2_] on the “base free” oxidation of glycerol

**Figure 5:**
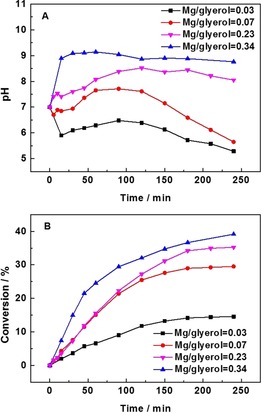
Conversion/pH vs. time profiles of glycerol oxidation over physically mixed (AuPd/C+Mg(OH)_2_). Graph A presents the pH evolution, while graph B presents the glycerol conversion with the increasing amount of Mg(OH)_2_. Reaction conditions: 1:1.85 mole fraction Au:Pd with 1 % metal loading by mass, water (35 mL), glycerol (0.3 m), mole fraction glycerol/metal=690, 60 °C, 4 h, 3 bar O_2_.

Additional glycerol oxidation tests were subsequently carried out using the physically mixed (AuPd/C+Mg(OH)_2_) catalyst with different Mg(OH)_2_/glycerol mole fractions and the results are shown in Figure 5 A,B. As is shown in Figure 5 A, the pH, as expected, increases gradually with the amount of Mg(OH)_2_. With a low Mg(OH)_2_/glycerol ratio (<0.23), the pH of the solution decreases in the first 15 min and then subsequently increases over the next 90 min of reaction, before starting to decrease rapidly. With a higher Mg(OH)_2_/glycerol molar ratio (0.23), the pH increases before 120 min reaction time and then decreases slightly in the following stages of the reaction. When the Mg(OH)_2_/glycerol reaches 0.34, the pH can be maintained at around pH 9 during the entire reaction. The corresponding data of the final Mg^2+^ concentration in the reaction solution is presented in Figure S4 A, and shows the same trend to [Mg(OH)_2_]. This result confirms the extent of the dissolution of Mg(OH)_2_ and that it can increase the pH of the solution and act to maintain the basicity of the reaction medium as the acidic products are produced.

The glycerol conversion as a function of time on line is presented in Figure 5 B. It is clear that the activity of the catalyst increases with increasing [Mg(OH)_2_], which corresponds to the pH observed. Davis and co‐workers showed that the oxygen source in the oxidation of glycerol over Au based catalysts is OH^−^.^21^ As such, it is rational to suggest that the concentration of OH^−^ could be directly related to the activity of AuPd nanoparticles, which is consistent with the observed phenomenon. As for the product distribution (Figure S4 B), the selectivity for DHA goes down gradually with the amount of Mg(OH)_2_, which is accompanied by an increasing selectivity towards acid products.

### Glycerol oxidation in the presence of NaOH and Mg(OH)_2_

We have shown that the pH evolution which occurs upon the addition of Mg(OH)_2_ is important for the performance of AuPd nanoparticles. To further explain the importance of the maintenance of pH on the catalytic performance, NaOH and Mg(OH)_2_ were added into the reaction solution during the reaction with the AuPd/C catalyst.

**Figure 6:**
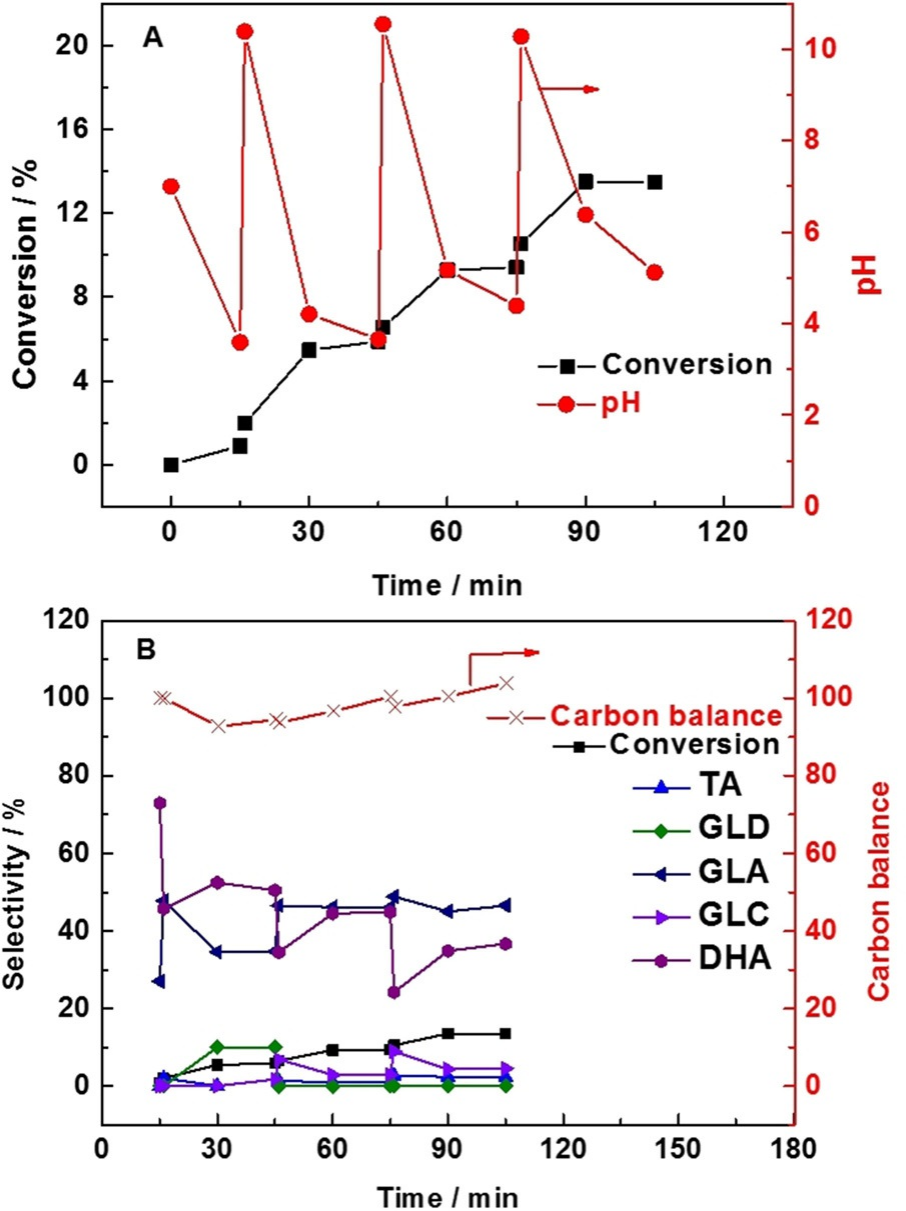
Glycerol oxidation in the presence of NaOH. TA (tartronic acid), GLA (glyceric acid), GLD (glyceraldehyde), GLC (glycolic acid), DHA (dihydroxyacetone). Reaction conditions: 1:1.85 mol fraction Au:Pd with 1 % metal loading by mass, water (35 mL), glycerol (0.3 m), mole fraction of glycerol/*meta l*=690, 60 °C, 4 h, 3 bar O_2_. NaOH (3 m) was added at the 15th, 45th and 75th min. The amount of NaOH added was 0.05, 0.06 and 0.05 mL, respectively, which correspond to 0.15, 0.18 and 0.15 mmol OH^−^.

Figure 6 shows the effect of NaOH addition during the reaction. During the first 15 min of reaction, only <1 % glycerol was converted and the pH decreased to 3.6. Once NaOH was added, an immediate increase of pH was observed (reaching 10), which resulted in a rapid increase in the oxidation of glycerol for the second 15 min (5.5 %). However, such small quantities of NaOH are not sufficient to stabilize and maintaing this elevated pH, owing to the increasing amount of acid products produced. The pH decreased to 4.2 at the end of the second 15 min period of reaction. In the following 15 min, the pH was kept at a very low value (4.2 to 3.7), resulting in only 0.4 % glycerol conversion. The same phenomenon could be observed as the second and third additions of NaOH were made. With regards to the product selectivity, it is clear that DHA is extremely sensitive to the increases in the pH, which is consistent with the previous observation that DHA is not stable at high pH.^18^ A marked decrease of DHA selectivity was observed after the addition of NaOH, leading to the rapid increase in the selectivity to glyceric acid.

**Figure 7:**
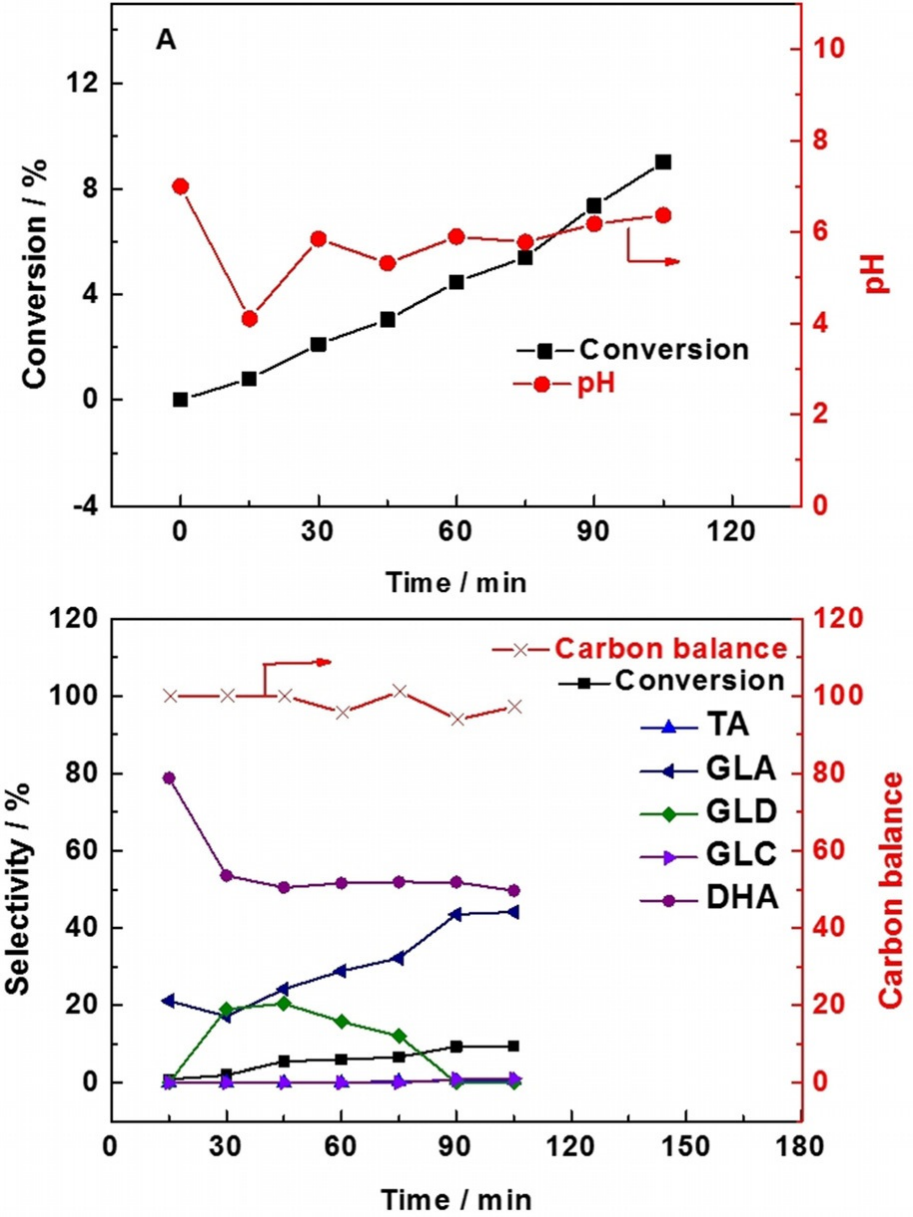
Glycerol oxidation in the presence of Mg(OH)_2_. TA (tartronic acid), GLA (glyceric acid), GLD (glyceraldehyde), GLC (glycolic acid), DHA (dihydroxyacetone). Reaction conditions: 1:1.85 mole fraction Au:Pd with 1 % metal loading by mass, water (35 mL), glycerol (0.3 m), mole fraction glycerol/metal=690, 60 °C, 3 bar O_2_, 4 h. Mg(OH)_2_ was added at the 15th, 45th and 75th min. The amount of Mg(OH)_2_ added was 4.4, 5.2 and 4.4 mg, respectively, which correspond to 0.15, 0.18 and 0.15 mmol OH^−^.

Figure 7 shows the performance of Mg(OH)_2_ addition with a concentration that corresponds to the same amount of OH^−^ provided by NaOH in the previous experiment. In contrast to the results observed with NaOH addition, the pH went up slowly and was maintained at around pH 6 during the 2 h reaction. However, with such a small amount of Mg(OH)_2_ at each time of addition (Mg(OH)_2_/glycerol<0.02), the pH is restricted by the rate of Mg(OH)_2_ dissolution. Consequently, the glycerol conversion increases gradually to 9 %, which is slightly lower than that observed with NaOH addition. Since the pH is maintained at a low level, a relatively high selectivity for DHA and low selectivity for glyceric acid is also observed.

These results explain the difference between Mg(OH)_2_ and NaOH with respect to the ability to adjust the pH. The dissolution of Mg(OH)_2_ can increase the pH of the solution, but its effect is restricted by the amount added. The rate of Mg(OH)_2_ dissolution, which is affected by the amount of Mg(OH)_2_ and the rate of OH^−^ consumption, which is controlled by the acid products, compete with each other and form the observed pH evolution. Unlike with the NaOH however, the highest pH for the solution in the presence of enough Mg(OH)_2_ will always be lower than 10.4, according to the solubility equilibrium at 25 °C.

### Effect of support on glycerol oxidation with NaHCO_3_

**Figure 8:**
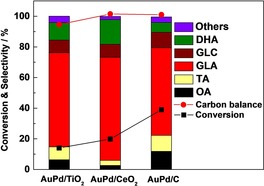
Effect of support on the performance of AuPd NPs in glycerol oxidation. Three kinds of catalysts were tested, AuPd/C, AuPd/TiO_2_, AuPd/CeO_2_. OA (oxalic acid), TA (tartronic acid), GLA (glyceric acid), GLC (glycolic acid), DHA (dihydroxyacetone). Conditions: 1:1.85 mol fraction Au:Pd with 1 % metal loading by mass, water (10 mL), glycerol (0.3 m), mole fraction glycerol/metal=690, 60 °C, 4 h, 3 bar O_2_, mole fraction NaHCO_3_/glycerol=3.

Mg(OH)_2_ when employed as a sacrificial support can maintain the pH at a relatively high level through its dissolution, while other supports (carbon, TiO_2_) could not stabilize the OH^−^ concentration once acidic products are produced. This is likely to be the main reason why Mg(OH)_2_ can attain such an outstanding performance under “base‐free” conditions (Figure S1). As such, it is not rational to compare the support effect, as the pH of the reaction media are different. Therefore, similar reactions were conducted with the addition of NaHCO_3_, to keep the solution at approximately pH 9. The results are shown in Figure 8.

For CeO_2_, TiO_2_ and carbon‐supported catalysts, a marked improvement in the catalytic activity occurs. The carbon‐supported catalyst gives the best activity in these conditions, which is approximately 39 % of glycerol conversion and even higher than that of catalyst Mg(OH)_2_. The activity of CeO_2_‐containing catalyst (20 % conversion) is better than AuPd supported on TiO_2_ (14 % conversion). However, without NaHCO_3_, they only convert 0.3 % and 1 % glycerol, respectively.

The product distribution varies among these supports. According to the reaction mechanism, high conversion should favour the highly oxidized products but CeO_2_‐supported catalyst shows lower selectivity than AuPd/TiO_2_ for tartronic acid, which is probably ascribed to the acid‐base nature of the support which has been discussed previously.^11^

## Conclusion

For a long time, the investigation into basic supports for the oxidation of alcohols has focused on correlating catalytic performance with their acid–base properties. Given that solid base supports are often sparingly soluble in an aqueous medium, it seems surprising that catalytic performance is often not associated with the formation of homogeneous base in solution. We have attempted to demonstrate that catalytic performance is exceptionally dependant on the pH of the aqueous medium. We have shown in this study that the activity of such catalysts is simply an effect of the dissolution of the basic support. We have discussed this in detail with respect to the influence of Mg(OH)_2_ on the pH of the solution during glycerol oxidation and compared it to the use of standard bases in similar quantities. We have shown the level of Mg(OH)_2_ dissolution by MP‐AES, and we have found that this can control the pH of the reaction solution, which can influence both the activity and selectivity of the reaction products. The rate of Mg(OH)_2_ dissolution, which is controlled by the amount of Mg(OH)_2_, competes with the rate of OH^−^ consumption from acid products. As a result, the pH will increase with the amount of Mg(OH)_2_, leading to the rise of AuPd activity and selectivity for acids. With enough Mg(OH)_2_, as the pH is not restricted by the Mg(OH)_2_ dissolution rate, the pH could be stabilized at a relatively high level. But, increasing Mg^2+^ concentration in turn affects the OH^−^ concentration according to the solubility equilibrium, leading to a gradual decline of the pH. These pH changes that Mg(OH)_2_ bring to the reaction system are in fact the main reason for the outstanding performance of AuPd NPs with Mg(OH)_2_ in the oxidation of glycerol. We believe that this is an important consideration when designing “base‐free” oxidation systems. We have shown that in reactions in water, especially when acid products are formed, magnesium based catalysts can never truly be described as base free.

## Experimental Section

**Materials**: KOH (laboratory reagent grade) and NaOH (>97 %) were purchased from Fisher Scientific UK Limited. PdCl_2_ and HAuCl_3_⋅3 H_2_O were purchased from Johnson Matthey. Polyvinyl alcohol (PVA) (M_w_=10 000, 80 % hydrolyzed), NaBH_4_, CeO_2_ (nanopowder, <25 nm particle size (BET)), and MgCl_2_ (>98 %, anhydrous) were purchased from Sigma–Aldrich. MgO was purchased from BDH Limited. TiO_2_ (Degussa P25) was purchased from Evonik. Carbon black (XC‐72R) is purchased from Cabot Corporation. The deionized water was purified by a Millipore water purification system.

**Mg(OH)_2_/C preparation**: Mg(OH)_2_ was deposited on the carbon via a precipitation method. Carbon black (4 g) was added to the solution of MgCl_2_ in deionized water (250 mL). After vigorous stirring for 2 h, aqueous solution of KOH (2 m) was added to adjust the pH to 12. The solid formed was recovered by filtration and washed with water until the pH of the filtrate decreased to 7, the solid was then dried (110 °C, 16 h).

**Catalyst preparation**: Au‐Pd nanoparticles were prepared and deposited on the supports using a sol‐immobilization method. Typically, the solution of HAuCl_3_⋅3 H_2_O (12.25 g in 1000 ml) and PdCl_2_ (1 g in 100 mL), polyvinyl alcohol (PVA) (1 wt % solution), NaBH_4_ (0.1 m) were prepared. Then, PVA (10 mg) was added into the aqueous solution of Au and Pd (Au/Pd mole fraction=1:1.85). After 5 min, NaBH_4_ (NaBH_4_/Metal mole fraction=4) solution was added to form a dark‐brown sol. After 30 min, support (1 g) was added to make the 1 wt % catalyst. The supports include Mg(OH)_2_, MgO, TiO_2_, Carbon black, CeO_2_ and mixed support Mg(OH)_2_/C (pre‐synthesized). After 2 h, the slurry was filtered and washed with water (250 mL) and then dried in 110 °C oven for 16 h.

**Glycerol oxidation**: The glycerol oxidation was carried out in a glass reactor (50 mL). Aqueous glycerol (0.3 m), and catalyst were added (mol fraction glycerol/metal=690). Then, pure oxygen was used to purge the reactor three times and then the oxygen pressure was maintained at 3 bar during the reaction. The reaction mixture was heated to 60 °C for 4 h and then cooled down in ice bath. The products were analyzed by high‐performance liquid chromatography (HPLC) with ultraviolet and refractive index detectors. A Metacarb 67H column was used to separate the products with 0.01 m aqueous H_3_PO_4_ at the rate of 0.8 mL min^−1^. The concentration of magnesium was measured by microwave plasma atomic emission spectroscopy (Agilent 4100 MP‐AES). The pH of the solution were all measured at room temperature with Mettler Toledo FE20‐Basic FiveEasy^TM^ Benchtop pH Meter.

**Characterization**: Powder X‐ray diffraction (XRD) was performed using a Panalytical X′pert Pro diffractometer using CuKα radiation at 40 kV and 40 mA. Scans were in the range of 10–80°. Transmission electron microscopy (TEM) was carried out using a Jeol 2100 with a LaB_6_ filament operating at 2000 kV. Ultra‐high spatial resolution EDX microanalysis was performed in a VG HB601‐UX scanning transmission electron microscope, operating at 100 kV.

## Conflict of interest

The authors declare no conflict of interest.

## Supporting information

As a service to our authors and readers, this journal provides supporting information supplied by the authors. Such materials are peer reviewed and may be re‐organized for online delivery, but are not copy‐edited or typeset. Technical support issues arising from supporting information (other than missing files) should be addressed to the authors.

Supplementary

## References

[1] G. J. Hutchings , C. J. Kiely , Acc. Chem. Res. 2013, 46, 1759–1772. 23586905 10.1021/ar300356m

[2] P. S. Kong , M. K. Aroua , W. Daud , Renewable Sustainable Energy Rev. 2016, 63, 533–555.

[3] A. Villa , N. Dimitratos , C. E. Chan-Thaw , C. Hammond , L. Prati , G. J. Hutchings , Acc. Chem. Res. 2015, 48, 1403–1412. 25884231 10.1021/ar500426g

[4] S. Carrettin , P. McMorn , P. Johnston , K. Griffin , G. J. Hutchings , Chem. Commun. 2002, 696–697. 10.1039/b201112n 12119680

[5] L. Prati , M. Rossi , J. Catal. 1998, 176, 552–560.

[6] N. Dimitratos , J. A. Lopez-Sanchez , D. Lennon , F. Porta , L. Prati , A. Villa , Catal. Lett. 2006, 108, 147–153.

[7] A. Villa , G. M. Veith , L. Prati , Angew. Chem. Int. Ed. 2010, 49, 4499–4502; 10.1002/anie.201000762 20461742

[8] S. A. Kondrat , P. J. Miedziak , M. Douthwaite , G. L. Brett , T. E. Davies , D. J. Morgan , J. K. Edwards , D. W. Knight , C. J. Kiely , S. H. Taylor , G. J. Hutchings , ChemSusChem 2014, 7, 1326–1334. 24955446 10.1002/cssc.201300834

[9] A. Villa , S. Campisi , K. M. H. Mohammed , N. Dimitratos , F. Vindigni , M. Manzoli , W. Jones , M. Bowker , G. J. Hutchings , L. Prati , Catal. Sci. Technol. 2015, 5, 1126–1132.

[10] G. L. Brett , Q. He , C. Hammond , P. J. Miedziak , N. Dimitratos , M. Sankar , A. A. Herzing , M. Conte , J. A. Lopez-Sanchez , C. J. Kiely , D. W. Knight , S. H. Taylor , G. J. Hutchings , Angew. Chem. Int. Ed. 2011, 50, 10136–10321; 10.1002/anie.201101772 PMC3644171 21990249

[11] C. L. Xu , Y. Q. Du , C. Li , J. Yang , G. Yang , Appl. Catal. B 2015, 164, 334–343.

[12] Z. F. Yuan , Z. K. Gao , B. Q. Xu , Chin. J. Catal. 2015, 36, 1543–1551.

[13] X. W. Han , L. Geng , Y. Guo , R. Jia , X. H. Liu , Y. G. Zhang , Y. Q. Wang , Green Chem. 2016, 18, 1597–1604.

[14] P. Patnaik , Handbook of Inorganic Chemicals, McGraw-Hill, New York, 2003.

[15] P. J. Miedziak , H. Alshammari , S. A. Kondrat , T. J. Clarke , T. E. Davies , M. Morad , D. J. Morgan , D. J. Willock , D. W. Knight , S. H. Taylor , G. J. Hutchings , Green Chem. 2014, 16, 3132–3141.

[16] M. Douthwaite , X. Huang , S. Iqbal , P. J. Miedziak , G. L. Brett , S. A. Kondrat , J. K. Edwards , M. Sankar , D. W. Knight , D. Bethell , G. J. Hutchings , Catal. Sci. Technol. 2017, 22, 5284–5293.

[17] M. S. Ide , R. J. Davis , Acc. Chem. Res. 2014, 47, 825–833. 24261465 10.1021/ar4001907

[18] E. G. Rodrigues , S. A. C. Carabineiro , J. J. Delgado , X. Chen , M. F. R. Pereira , J. J. M. Orfao , J. Catal. 2012, 285, 83–91.

[19] C. Appayee , R. Breslow , J. Am. Chem. Soc. 2014, 136, 3720–3723. 24575857 10.1021/ja410886c

[20] L. S. Sharninghausen , J. Campos , M. G. Manas , R. H. Crabtree , Nat. Commun. 2014, 5, 5084. 25278373 10.1038/ncomms6084

[21] B. N. Zope , D. D. Hibbitts , M. Neurock , R. J. Davis , Science 2010, 330, 74–78. 20929807 10.1126/science.1195055

