# Supplementary material for: The Role of Mg(OH)2 in the So‐Called “Base‐Free” Oxidation of Glycerol with AuPd Catalysts
Source: Chemistry. 2018 Jan 24;24(10):2396–402. doi: 10.1002/chem.201704151 (PMC5969100; doi:10.1002/chem.201704151)
Supplement: Supplementary file 1 — Supplementary [file CHEM-24-2396-s001.pdf]

CHEMISTRY
A European Journal

Supporting Information

The Role of $Mg(OH)_2$ in the So-Called "Base-Free" Oxidation of Glycerol with AuPd Catalysts

Jile Fu+,[a, b] Qian He+,[a] Peter J. Miedziak,[a] Gemma L. Brett,[a] Xiaoyang Huang,[a] Samuel Pattisson,[a] Mark Douthwaite,[a] and Graham J. Hutchings*[a]

chem_201704151_sm_miscellaneous_information.pdf

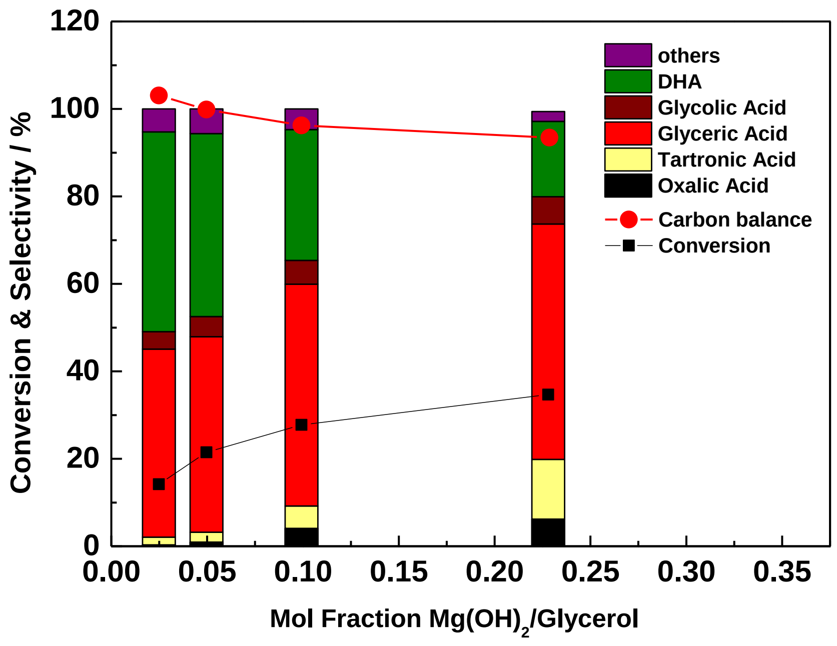

Figure S1. Effect of the amount of $Mg(OH)_2$ on the performance of AuPd-$Mg(OH)_2$/C in glycerol oxidation. OA (oxalic acid), TA (tartronic acid), GLA (glyceric acid), GLD (Glyceraldehyde), GLC (glycolic acid), DHA (dihydroxyacetone). Reaction conditions: 1:1.85 mole fraction Au: Pd with 1% metal loading by mass, water (10 mL), glycerol (0.3 M), mole fraction glycerol/metal = 690, 60 °C, 4 h, 3 bar O_2.

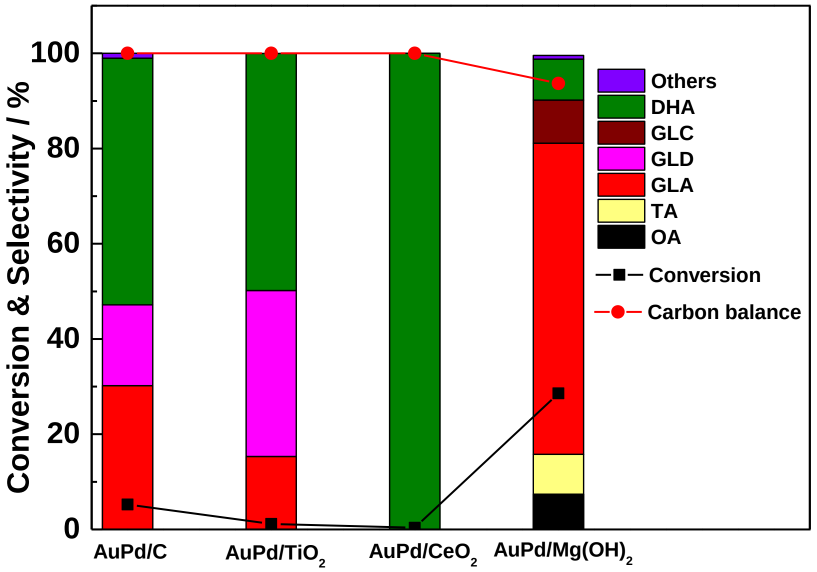

Figure S2. Effect of support on the performance of AuPd NPs in glycerol oxidation. Catalysts tested are AuPd/C, AuPd/TiO_2, AuPd/CeO_2 and AuPd/$Mg(OH)_2$. OA (oxalic acid), TA (tartronic acid), GLA (glyceric acid), GLD (Glyceraldehyde), GLC (glycolic acid), DHA (dihydroxyacetone). Reaction conditions: 1:1.85 mole fraction Au: Pd with 1% metal loading by mass, water (10 mL), glycerol (0.3 M), mole fraction glycerol/metal = 690, 60 °C, 4 h, 3 bar O_2.

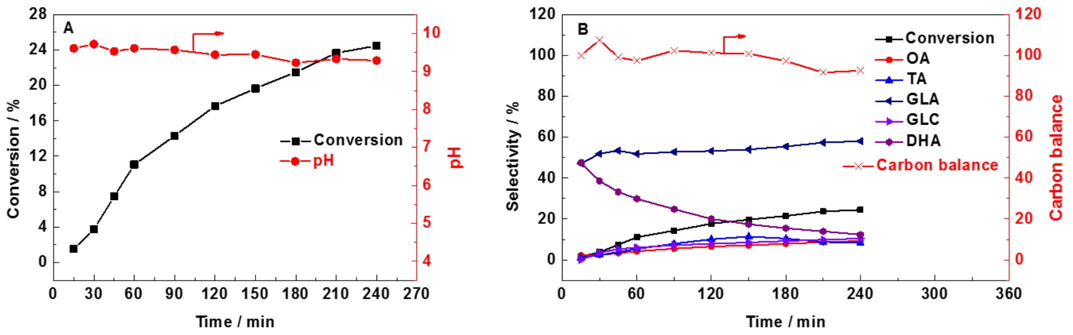

Figure S3. Conversion/selectivity/pH/carbon balance vs time profiles of glycerol oxidation over AuPd/$Mg(OH)_2$. OA (oxalic acid), TA (tartronic acid), GLA (glyceric acid), GLC (glycolic acid), DHA (dihydroxyacetone). Conditions: 1:1.85 mole fraction Au: Pd with 1% metal loading by mass, water (35 mL), glycerol (0.3 M), mole fraction glycerol/metal = 690, mole fraction $Mg(OH)_2$/glycerol = 0.34, 60 °C, 4 h, 3 bar O_2.

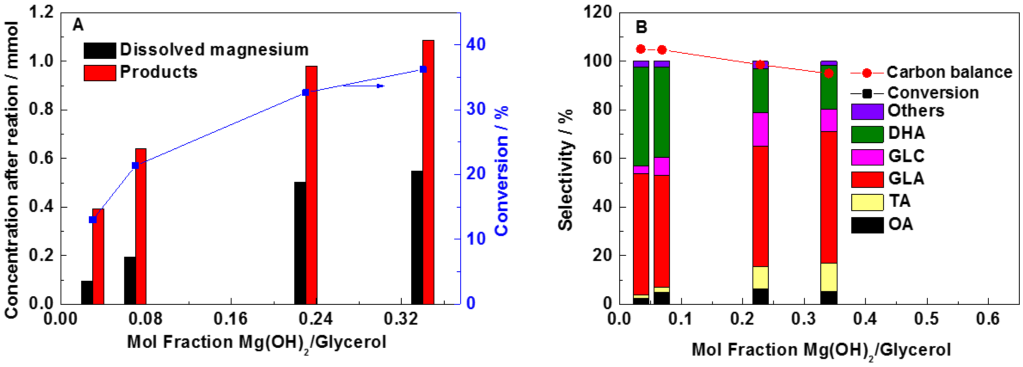

Figure S4. Comparison of the amount of magnesium and products in the solution after reaction over physically mixed materials (AuPd/C+$Mg(OH)_2$). Graph A shows the magnesium concentration in the solution after a reaction with different amounts of $Mg(OH)_2$. Graph B compares the product distribution with different amounts of $Mg(OH)_2$. OA (oxalic acid), TA (tartronic acid), GLA (glyceric acid), GLC (glycolic acid), DHA (dihydroxyacetone). Reaction conditions: 1:1.85 mole fraction Au: Pd with 1% metal loading by mass, water (35 mL), glycerol (0.3 M), mole fraction glycerol/metal = 690, 60 °C, 4 h, 3 bar O_2.
